# Treatment of sleep apnoea with tonsillectomy: a retrospective analysis using long-term follow-up data

**DOI:** 10.1007/s00405-022-07350-6

**Published:** 2022-03-25

**Authors:** Henrik M. Sjöblom, Max Nahkuri, Miika Suomela, Jussi Jero, Jaakko M. Piitulainen

**Affiliations:** 1 Department of Otorhinolaryngology, Head and Neck Surgery, Division of Surgery and Cancer Diseases, Turku University Hospital, POB 52, 20521 Turku, Finland; 2 Department of Clinical Neurophysiology, Turku University Hospital, Turku, Finland; 3 Department of Medicine, University of Turku, Turku, Finland; 4 Department of Medicine, University of Helsinki, Helsinki, Finland

**Keywords:** Adult tonsillectomy, Obstructive sleep apnoea, Sleep apnoea syndromes, Adult tonsillar hypertrophy

## Abstract

**Purpose:**

This single-group, retrospective, pre-test–post-test study was performed to examine clinical outcomes in treating obstructive sleep apnoea (OSA) with tonsillectomy alone and had the longest follow-up periods to date.

**Methods:**

We analysed 151 tonsillectomies in our district between the years 2004 and 2018 that had either sleep apnoea or snoring listed as a diagnosis. Twenty-one patients met our criteria and were included. Patient records were analysed for home sleep apnoea test and Epworth Sleepiness Scale (ESS) scores.

**Results:**

We defined success as a > 50% reduction of the Apnoea–Hypopnea Index (AHI) and a total AHI of < 20 post-surgery. The averages before surgery were an AHI of 22.3 and an ESS of 7.22. The success rate was 47.6% after tonsillectomy as the sole treatment for obstructive sleep apnoea in our adult population. Eleven patients were non-responders. The average ESS score reduction was 0.69 and did not reach statistical significance. With follow-up times ranging from 1.8 to 171 months, this study had the longest follow-up period compared to other existing studies. No patient with a follow-up longer than one year was a responder.

**Conclusion:**

Our results support that tonsillectomy is an effective treatment for obstructive sleep apnoea in adults with tonsillar hypertrophy. With less severe OSA than those reported on previously, our patients also had less severe daytime sleepiness before surgery, and daytime sleepiness score reductions did not reach statistical significance. In the future, long-term results should be further analysed.

**Supplementary Information:**

The online version contains supplementary material available at 10.1007/s00405-022-07350-6.

## Introduction

Obstructive sleep apnoea (OSA) causes snoring and excessive daytime sleepiness leading to many patients to seek treatment. Continuous positive airway pressure (CPAP) is a highly effective treatment for many, but 46%–83% of patients are non-adherent to therapy [1]. Numerous different surgery techniques have been described for the treatment of OSA [2] with a large variability in outcomes. Uvulopalatopharyngoplasty, one of the first surgical treatments, is a commonly used intervention [3] even though it involves muscular resection and up to 50% of patients are not satisfied with the result of the operation after 20 years [4]. There is a debate to the effectiveness of the general surgical treatment altogether for OSA. Thirteen per cent of CPAP-treated patients also have residual excessive sleepiness [5]. Residual sleepiness is nearly twice as prevalent in patients suffering from moderate OSA when compared to patients with severe OSA [5, 6]. CPAP treatment elicits only small improvements in subjective daytime sleepiness scores in patients with mild to moderate OSA [6].

Isolated tonsillectomy is a treatment option for adult obstructive sleep apnoea (OSA), especially amongst patients with large tonsils and moderate OSA with an Apnoea–Hypopnea Index (AHI) of less than 30 per hour. In cases of tonsillar hypertrophy, published findings suggest that soft palate surgery has no significant added impact on surgical success [7]. For paediatric patients with OSA, adenotonsillectomy is the first-line treatment [8]. A review from year 2016 found 17 studies that reported on tonsillectomy alone in the treatment of adult sleep apnoea with four being prospective [9]. Since then, two other prospective studies have been published with promising results [10, 11]. Patient numbers in reports range from 1 to 56. Reported follow-up times range from 1 to 15 months and, in most of the studies, they are 6 months or less.

Up to 13% of men and 6% of women are estimated to have moderate to severe OSA [12]. Since data available for such a common operation for the treatment of such a frequently occurring condition are scarce, we aimed here to further the knowledge on this subject.

## Materials and methods

This is a single-group, retrospective, pre-test–post-test study. In this study, we analysed all the tonsil and adenoid surgeries performed in the hospital district of Southwest Finland between the years 2004–2018. Of those surgeries, we limited the results to those aged 16 or older, classified here as adults, and surgeries which only contained the Nordic classification code for tonsillectomy (EMB10) along with the diagnosis codes of either sleep apnoea (G47.3) or snoring (R06.5).

Patients’ age, weight and height were gathered, and body mass index (BMI) was calculated from the results when sufficient data were available. Also, retrospective data from home sleep apnoea tests were used. We also recorded Epworth Sleepiness Scale (ESS) points when available. We gathered pre- and post-surgery data where available. We analysed whether the patient had undergone tonsillectomy alone or had simultaneous surgeries along with it. We also analysed any associated nasal pathologies.

Patients who did not have both pre- and post-surgery home sleep apnoea tests available were excluded. Only patients who had undergone a tonsillectomy with or without only a partial uvulectomy were included in the analysis. Statistical analysis was carried out using the JMP Pro 15.1.0 software (SAS Institute Inc., Cary, NC, USA).

## Results

**Fig. 1:**
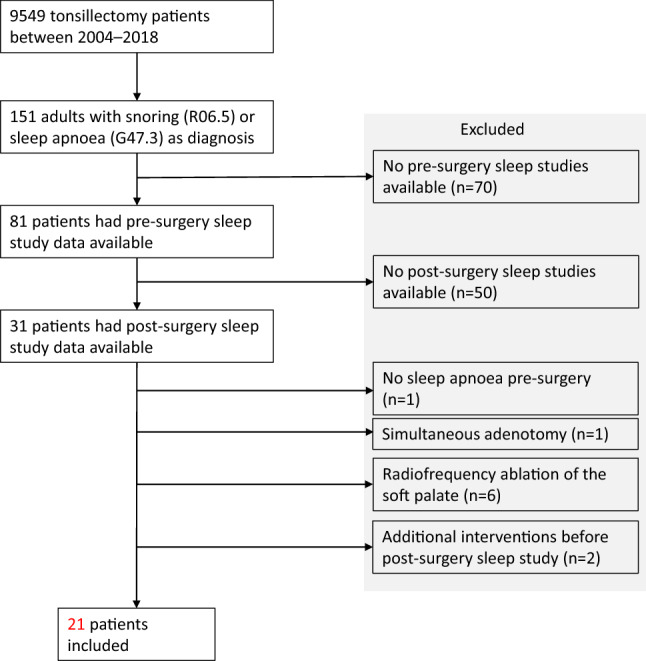
Study selection flow chart

A total of 9549 patients in our hospital district underwent tonsillectomy between 2004 and 2018. One hundred and fifty one surgeries on adults had either sleep apnoea or snoring listed as a diagnosis. Of these patients, 81 had pre-surgery sleep studies available and 31 of those had post-surgery sleep studies available. When reviewing the patient records, we noted that 6 patients had simultaneous radiofrequency ablation of the soft palate and one had simultaneous adenotomy and all of these 7 were excluded. Eight patients had a simultaneous partial uvulectomy in the same surgery and were included in our analysis. One of the patients had no sleep apnoea prior to surgery (AHI 1.9) and was excluded. Two patients had undergone additional treatments before the post-surgery sleep study had been completed and were excluded, as one patient underwent a palatoplasty operation after the tonsillectomy and one patient had a dental device fitted and used it in the follow-up sleep study. A total of 21 patients were included in this study. See Fig. 1 for the study selection flow chart.

Individual results of each patient are shown in Table 1, including tonsil size, pre- and post-operative AHI values, AHI change, BMI before surgery, weight change and time from surgery to their first sleep study.

**Table 1:** Individual results of each patient and some notes on additional findings sorted into two groups by ascending tonsil size estimated from written descriptions

Patient ID	Tonsil size description	Pre-op AHI	Post-op AHI	AHI change	AHI change (%)	BMI pre-op	Weight change (%)	Time from surgery to sleep study (months)	Notes
12	Small	21	9	− 12.0	− 57.1%	27.1	N/A	3.5	
8	Medium	49.3	17.6	− 31.7	− 64.3%	26.1	+ 3.8%	8.9	
15	Medium	7.7	5.8	− 1.9	− **24.7%**	24.6	+ 9.3%	87.5	
19	Medium	16.9	7.2	− 9.7	− 57.4%	35.1	+ 2.8%	6.2	*
Avg	Small– Medium	23.73	9.9	− 13.83	− 50.88%				
1	Large	24	5.10	− 18.9	− 78.8%	25.6	+ 3.5%	3.3	
4	Large	44.7	56	11.3	** + 25.3%**	40.4	+ 29.7%	149.5	
5	Large	20	3.2	− 16.8	− 84.0%	24.8	0.0%	6.9	
6	Large	11.7	3.9	− 7.8	− 66.7%	26.3	+ 3.8%	8.1	
9	Large	9	5.1	− 3.9	− **43.3%**	29.1	+ 4.5%	2.6	
10	Large	25.7	3.8	− 21.9	− 85.2%	29.4	− 3.1%	3.6	**
11	Large	9.4	**21.2**	11.8	** + 125.5%**	26.6	+ 10.9%	171.0	
14	Large	33.8	1.5	− 32.3	− 95.6%	25.9	+ 0.4%	6.1	
16	Large	10.4	4.6	− 5.8	− 55.8%	29.9	− 2.0%	2.2	
17	Large	6.4	12.2	5.8	** + 90.6%**	29.4	+ 9.9%	98.7	
20	Large	31.7	4.1	− 27.6	− 87.1%	25.5	+ 1.2%	5.9	
21	Large	4.9	10	5.1	** + 104.1%**	31.8	+ 8.5%	25.0	
2	Very Large	54.5	17	− 37.5	− 68.8%	N/A	N/A	1.9	
3	Very Large	33.8	2.4	− 31.4	− 92.9%	27.7	0.0%	1.8	***
7	Very Large	35	**23.4**	− 11.6	− **33.1%**	27.8	+ 0.4%	6.6	
13	Very Large	8.5	16	7.5	** + 88.2%**	32.8	+ 5.2%	164.3	
18	Very Large	9.8	0.9	− 8.9	− 90.8%	23.6	+ 22.5%	6.0	
Avg	Large– Very Large	21.96	11.2	− 10.76	− 26.37%				

Unfavourable outcomes are marked with bold (AHI > 20 and/or < 50% reduction of AHI post-operatively). N/A = not available

^*^Four years after surgery, the patient became symptomatic again and CPAP treatment re-started

^**^AHI 9.1 ten years after surgery and has since re-started CPAP treatment

^***^AHI 38.3 nine years after surgery and has since re-started CPAP treatment

Tonsil sizes were translated to English from Finnish descriptions and sorted into 4 categories: small, medium, large and very large. Numeric (e.g. Friedman–Brodsky) tonsil grades were not available for any patient in our retrospective records. Five patients had very large, 12 had large, 3 had medium and 1 had small tonsils, respectively. The mean BMI before surgery was 28.48 (95% CI [26.58–30.37]). The weight changes, ranging from -3.1% to + 29.7% in individual patients, are shown in Table 1.

The first post-surgery sleep studies were performed an average of 1099 days after surgery (95% CI [298–1901 days]). The shortest duration was 53 days after surgery, and the longest was 5131 days or a little over 14 years. In our clinic, during the examined period, a post-surgery test was not routine and was most often performed because of residual symptoms.

**Fig. 2:**
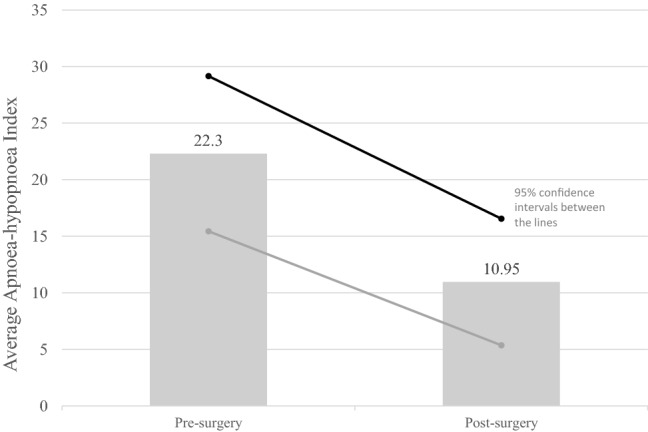
Average AHI of patients pre- and post-tonsillectomy. 95% confidence intervals between the lines

**Fig. 3:**
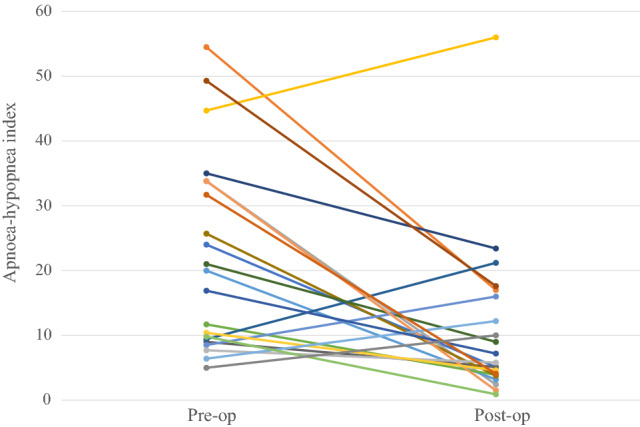
Pre- and post-tonsillectomy AHI of each patient individually

**Fig. 4:**
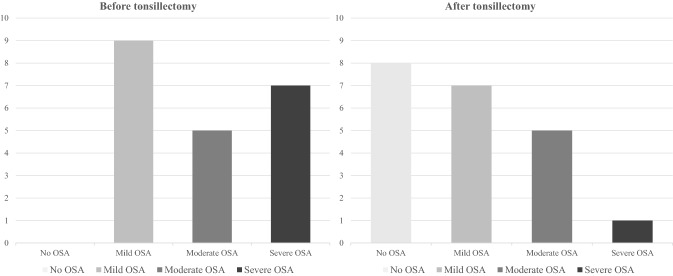
The distribution of OSA severity amongst patients pre- and post-tonsillectomy

The average AHI before tonsillectomy was 22.3, and the post-operative average was 10.95 (Fig. 2, *P* = 0.0026). Figures 3 and 4 show the distribution of OSA severity amongst patients pre- and post-surgery. Before surgery, nine (43%), five (24%) and seven (33%) patients had mild, moderate and severe OSA, respectively, and these were classified by an AHI < 15, an AHI 15–30 and an AHI ≥ 30, respectively. After tonsillectomy, eight patients no longer had sleep apnoea (38%), seven patients had mild OSA (33%), five patients had moderate OSA (24%) and one patient had severe OSA (5%). The patient (ID 4) with severe OSA after surgery had a significant weight gain of 29.7%. Of the nine patients with mild OSA before surgery, four still had mild OSA and two progressed into moderate OSA after surgery.

We defined surgical success as the AHI decreasing by > 50% and a total AHI of < 20 post-surgery. The initial success rate was 61.9%, and 8 patients were non-responders. As stated above, a complete surgical cure (AHI < 5) was achieved in 38.1% (8 patients). In the small subgroup of patients with small- or medium-sized tonsils (*n* = 4), the success rate was 75%, though none were surgically cured (Table 1). No patient having a follow-up a year or more after the surgery was a success. After reviewing patient charts, we found 3 of the initial responders have since started CPAP treatment again and the true success rate should be considered 47.6%. One of the four patients with a BMI over 30 pre-surgery was a surgical success.

Pre- and post-operative daytime sleepiness measured with the Epworth Sleepiness Scale was available for 18 and 17 patients, respectively. It was very slightly reduced from a mean of 7.22 points (95% CI [5.51–8.93]) to 6.53 (95% CI [5.01–8.05]) at the time of each patient’s respective post-surgery sleep study (*P* = 0.2237).

Thirteen patients were operated on with cold instruments and 8 with electrocautery. Operations were performed by specialists (39.1%) and residents (61.9%). One patient had primary bleeding within 24 h of surgery and 3 (14.3%) had secondary post-tonsillectomy haemorrhage. No other complications occurred. Two patients underwent a septoplasty without any positive effect on the home sleep study AHI values before tonsillectomy. No other significant nasal pathologies were reported.

## Discussion

We defined success as the AHI decreasing by > 50% and a total AHI of < 20 post-surgery, which is similar to other studies [10, 13]. The true success rate was 47.6% after tonsillectomy as the sole treatment for obstructive sleep apnoea in our adult population. Eight patients were initial non-responders and three additional patients reverted to CPAP treatment later and were thus also counted as failures in this study. To the best of our knowledge, with follow-up times ranging from 1.8 to 171 months with a mean of 36.6 months and a median of 6.2, when compared to other related studies, this study had the longest follow-up period. The average reduction in daytime sleepiness, 0.69 points in the ESS, was in line with other interventions reducing the AHI [6], but the results did not reach statistical significance. These results are underscored by a selection bias more likely favouring patients with residual symptoms.

Notably, the patients with the six longest follow-up intervals were all non-responders having follow-up times of two years or longer. Additionally, whilst reviewing patient charts, we found that 3 of the responders, who stopped using CPAP treatment after surgery, have since started CPAP treatment again, usually some years after surgery. Two primary non-responders had respectively substantial weight gains of 10.9% and 29.7% post-surgery.

All but four of our patients had large or very large tonsils preoperatively. Most prospective studies have only recruited patients with tonsils having a grade 3 or 4. To our knowledge, only two prospective trials have included patients with grade 2 tonsils. Senchak et al. [14] reported of 2 patients with grade 2 tonsils who responded well to surgery. Nakata et al. [15] reported on 7 patients with grade 2 tonsils of whom 3 were responders (42.9%). Interestingly, in our study, the only patient with small tonsils was a responder, and 2 of the 3 patients with medium tonsils were responders. Because of the retrospective nature of this study, we cannot conclude exact tonsil grades. However, we speculate that tonsils described as small or medium are most likely grade 2 or smaller. When grouped, patients with small to medium tonsils had an average AHI change of -13.8 (-50.9%) compared to -10.8 (-26.4%) in those with large to very large tonsils. As such, the effect of tonsillectomy on OSA patients with grade 2 tonsils should be studied more.

Before surgery, our patients had a less severe OSA with an AHI of 22.3 compared to previous studies with an average AHI of 31.6–40.6 [9–11]. This, along with our relatively long follow-up times, might be reflected in our relatively low success rate of 47.6% compared to those of up to 73.5%–94.7% reported previously [14, 16]. In our study, the patients with mild OSA before surgery had, at best, a very modest benefit. It has been speculated that patients with less severe OSA might have multifactorial sleepiness and, as such, will not respond favourably to treatment of OSA alone [5]. Previous studies have had good results with tonsillectomy alone in patients with large tonsils. In these carefully selected patients, Holmlund et al. reported the mean AHI to decrease from 40 units per hour to 7 units per hour and Smith et al. stated the mean AHI decreased from 31.6 units to 8.1 units. These findings reinforce the importance of patient selection in OSA surgery. We would like to see future studies comparing patients with mild OSA to those with moderate OSA since they are analysed as a group with an AHI of less than 30 group in most published studies [9].

The less severe OSA of our patients might also be reflected in less of a response in reduced daytime sleepiness. The previously mentioned studies saw a significant reduction as measured by ESS. Our post-operative ESS value of 6.53 is comparable to previously reported post-operative values of 5.0–6.1, but the pre-operative ESS of 7.22 is significantly lower than, for example, the 11.6 (± 3.7) reported in the systematic review and meta-analysis published by Camacho et al. [9]. The ESS scores of normal controls are 5.9 ± 2.2 with a range of 2–10 [17]. It may not have an equal benefit on patients’ daytime sleepiness to operate on milder OSA, even though tonsillectomy might reduce the AHI measured in sleep studies successfully. For comparison, CPAP treatment has similarly been found to reduce ESS by an average of just 1.2 points in patients with mild to moderate OSA (AHI 5–30) [6].

The mean BMI before surgery in our study was comparably low at 28.48. Previous studies suggest that tonsillectomy may be successful in treating OSA in patients with a BMI of up to 35 [15]. In our study, only one of the four patients with a pre-surgery BMI of over 30 was a surgical success.

Eight patients underwent a partial uvulectomy in addition to tonsillectomy. As an uvulectomy has no positive long-term effect on OSA [18], we decided to include these patients in the analysis. As always with retrospectively collected data, we are dependent on chart records. Tonsil size was derived from verbal description. Modified Mallampati values or Friedman–Brodsky stages were not available. For weight, we relied on the values listed in sleep study reports. We were unable to find any post-tonsillectomy sleep studies for 50 patients with pre-surgery sleep studies available in our data, as a post-operative sleep study was not routinely done in our clinic earlier. It is possible that patients with positive long-term outcomes have remained symptomless, and, as such, have not needed new sleep studies since. These patients should be examined in a future study.

Uvulopalatopharyngoplasty results have been reported to drop significantly 6–12 months after surgery [19]. Most reported tonsillectomy outcomes in the treatment of OSA have a follow-up period of 6 months or less [9–11]. The limited number of patients in this study with long-term data available were non-responders, and three primary responders have since reverted to CPAP treatment. More research on long-term results is warranted.

In a follow-up study, we will gather new home sleep study results from additional willing patients from whom we only have pre-surgery sleep studies available and more thoroughly review the long-term results of tonsillectomy in the treatment of sleep apnoea 5 to 20 years post-surgery.

## Conclusion

Our results reinforce that tonsillectomy seems to be an effective treatment for obstructive sleep apnoea in adults with tonsillar hypertrophy. Significant reductions in AHI values are likely to extend to at least 6 months post surgery. Our patients with less severe OSA than those reported on previously had an average normal daytime sleepiness even before surgery and the reduction of daytime sleepiness scores did not reach statistical significance.

## Supplementary Information

Below is the link to the electronic supplementary material.

Supplementary file1 (PDF 51 kb)

## Data Availability

Data of each patient are presented in Table 1.

## Code availability

Statistical analysis was carried out using the JMP Pro 15.1.0 software (SAS Institute Inc., Cary, NC, USA).
