# Supplementary material for: Treatment of sleep apnoea with tonsillectomy: a retrospective analysis using long-term follow-up data
Source: Eur Arch Otorhinolaryngol. 2022 Mar 25;279(7):3727–32. doi: 10.1007/s00405-022-07350-6 (PMC9130194; doi:10.1007/s00405-022-07350-6)
Supplement: Supplementary file 1 — Supplementary file1 (PDF 51 kb) [file 405_2022_7350_MOESM1_ESM.pdf]

Regional Medical Research Ethics Committee
Hospital District of Southwest Finland
P.O. Box 52, FI-20521 Turku, Finland
Email:Eettinen.toimikunta@tyks.fi

To whom it may concern,

This letter is intended to clarify when an ethical committee review is required in health research in Finland and when it is not.

A statement is required:
Finnish law requires review by a statutory medical research ethics committee for all research, which interferes with the integrity of a person, human embryo, or human foetus, and which seeks to increase the understanding of health, the causes, symptoms, diagnosis, treatment and prevention of diseases, or the nature of diseases in general. Statutory committees include the regional medical research ethics committees of hospital districts and the National Committee on Medical Research Ethics, which reviews pharmaceutical trials only.

A statement is usually required also for research using already collected human biological material, unless based on informed consent which covers the intended research.

A statement is not required for example for:
- desk studies and register-based studies (even if using patient data),
- interviews and surveys, unless deemed invasive (like psychologically strenuous or disturbing interviews), or
- observational studies, unless they affect patient care (for example require randomization to compare treatment options).

Primary relevant legislation (available from www.finlex.fi):
- Medical Research Act (488/1999, as amended),
- Act of the Medical Use of Human Organs, Tissues and Cells (101/2001, as amended),
- Biobank Act (688/2012, as amended).

Hospital District of Southwest Finland Research Ethics Committee
